# Increased Lymphangiogenesis and Lymphangiogenic Growth Factor Expression in Perivascular Adipose Tissue of Patients with Coronary Artery Disease

**DOI:** 10.3390/jcm8071000

**Published:** 2019-07-09

**Authors:** Ioannis Drosos, Maria Pavlaki, Maria Del Pilar Ortega Carrillo, Adriani Kourkouli, Katja Buschmann, Fotios Konstantinou, Rajinikanth Gogiraju, Magdalena L. Bochenek, Georgios Chalikias, Christos Tortopidis, Christian F. Vahl, Dimitrios Mikroulis, Dimitrios Tziakas, Thomas Münzel, Stavros Konstantinides, Katrin Schäfer

**Affiliations:** 1 Center for Cardiology, Cardiology 1, University Medical Center of the Johannes Gutenberg University Mainz, 55131 Mainz, Germany; 2 Department of Cardiology, Democritus University of Thrace, 68100 Alexandroupolis, Greece; 3 Department of Cardiothoracic and Vascular Surgery, University Medical Center of the Johannes Gutenberg University Mainz, 55131 Mainz, Germany; 4 Department of Cardiothoracic Surgery, Democritus University of Thrace, 68100 Alexandroupolis, Greece; 5 Center for Thrombosis and Hemostasis, University Medical Center of the Johannes Gutenberg University Mainz, 55131 Mainz, Germany

**Keywords:** coronary artery disease, human, inflammation, lymphangiogenesis, perivascular adipose tissue

## Abstract

Experimental and human autopsy studies have associated adventitial lymphangiogenesis with atherosclerosis. An analysis of perivascular lymphangiogenesis in patients with coronary artery disease is lacking. Here, we examined lymphangiogenesis and its potential regulators in perivascular adipose tissue (PVAT) surrounding the heart (C-PVAT) and compared it with PVAT of the internal mammary artery (IMA-PVAT). Forty-six patients undergoing coronary artery bypass graft surgery were included. Perioperatively collected C-PVAT and IMA-PVAT were analyzed using histology, immunohistochemistry, real time PCR, and PVAT-conditioned medium using cytokine arrays. C-PVAT exhibited increased PECAM-1 (platelet endothelial cell adhesion molecule 1)-positive vessel density. The number of lymphatic vessels expressing lymphatic vessel endothelial hyaluronan receptor-1 or podoplanin was also elevated in C-PVAT and associated with higher inflammatory cell numbers, increased intercellular adhesion molecule 1 (ICAM1) expression, and fibrosis. Significantly higher expression of regulators of lymphangiogenesis such as vascular endothelial growth factor (VEGF)-C, VEGF-D, and VEGF receptor-3 was observed in C-PVAT compared to IMA-PVAT. Cytokine arrays identified angiopoietin-2 as more highly expressed in C-PVAT vs. IMA-PVAT. Findings were confirmed histologically and at the mRNA level. Stimulation of human lymphatic endothelial cells with recombinant angiopoietin-2 in combination with VEGF-C enhanced sprout formation. Our study shows that PVAT surrounding atherosclerotic arteries exhibits more extensive lymphangiogenesis, inflammation, and fibrosis compared to PVAT surrounding a non-diseased vessel, possibly due to local angiopoietin-2, VEGF-C, and VEGF-D overexpression.

## 1. Introduction

Accumulating clinical and experimental data suggest an active contribution of the adventitia to remodeling processes in the vascular wall, including neointima formation and atherosclerosis. For example, preclinical studies in hypercholesterolemic animal models have shown that adventitial angiogenesis promotes atherosclerotic plaque progression, whereas inhibition of plaque neovascularization reduced lesion growth [1,2,3]. The expansion of thin-walled, leaky vasa vasorum and microvessels within atherosclerotic lesions has been pathophysiologically linked to plaque instability by increasing inflammatory cell infiltration and intraplaque hemorrhage [4,5,6], among others.

In addition to arteries and veins, lymphatic vessels exist and develop in parallel with blood vessels in most organs. Although the extent and complexity of the lymphatic vasculature has been known for more than a century [7], its role in the pathogenesis of cardiovascular disease processes has received little attention. Lymph vessels control the drainage of interstitial fluids and macromolecules (proteins, lipids) leaking from capillaries back to the venous circulation and are thus critical for the maintenance of tissue homeostasis [8]. In addition, they have an important function in immune surveillance and may accelerate the resolution of inflammation by removing inflammatory mediators or trafficking immune cells to draining lymph nodes. Lymph vessel dysfunction results in lymphedema, but also may play a role during inflammation, cancer, obesity, or hypertension (reviewed in [9]).

Regarding atherosclerosis, which is characterized by chronic inflammation and cholesterol accumulation in the arterial wall, the possibility of a direct contribution of adventitial lymphangiogenesis has only been recently addressed. Studies in animal models after experimentally induced lymphostasis or genetic modulation of lymphatic vessel formation suggested a protective role of the lymphatic vasculature during atherosclerosis [10,11]. Moreover, cardiac lymphangiogenesis has been shown to take part in the clearance of immune cells and resolution of inflammation after myocardial infarction [12,13]. A causal role of lymph vessel dysfunction in the pathophysiology of atherosclerosis is further supported by findings in mice that excessive cholesterol accumulation is associated with structural and functional alterations of lymphatic vessels [14], and that lymphatic dysfunction develops before atherosclerotic lesion formation [15]. As far as human atherosclerosis is concerned, the available knowledge is limited to autopsy studies of large arteries from organ donors and the clinical observation that (adventitial) lymphangiogenesis correlates with atherosclerosis severity [11,16,17,18]. Similar findings were obtained in 21 endarterectomy samples from patients with internal carotid artery stenosis showing a more developed lymphatic network in the adventitia of atherosclerotic lesions compared to healthy arteries [19]. Whether these observations reflect lymphatic dysfunction or the reactive enlargement of perivascular lymphatic networks to local cues or systemic differences between patients at risk and control individuals remains unknown.

Given the anatomical proximity of perivascular adipose tissue (PVAT) with the adventitia and its known paracrine activities on the underlying vessel, studied by our group [20] and others (reviewed in [21]), we hypothesized that differences may exist in the extent of the lymphatic vasculature and the expression of lymphangiogenic growth factors between PVAT surrounding the aortic root and coronaries of patients with coronary artery disease (CAD) and PVAT of the internal mammary artery (IMA), an artery protected from the development of atherosclerosis [22].

## 2. Materials and Methods

### 2.1. Study Patients

Forty-six patients (76% male; mean age, 68.6 ± 9.8 years; mean body mass index, 29.1 ± 5.4 kg/m^2^) undergoing elective coronary artery bypass graft surgery in two university medical centers were included in the study. All patients had been diagnosed with multivessel CAD via coronary angiography. The left (and in some cases also the right) IMA was used as an aortocoronary bypass in all patients. The study complied with the declaration of Helsinki and was approved by the Ethics Committee of the University General Hospital of Alexandroupolis, Greece, and the University Medical Center of the Johannes Gutenberg University Mainz, Germany, respectively. All patients signed a written informed consent prior to inclusion in the study. Due to the limited amount of tissue from each patient, not all analyses could be performed in all patients, and samples had to be split into groups.

### 2.2. Tissue Specimen Collection

‘Cardiac’ perivascular adipose tissue (C-PVAT), located adjacent to the aortic root and around the coronary arteries, and PVAT surrounding the IMA (IMA-PVAT) was collected perioperatively and immediately transferred to the laboratory in ice-cold sterile saline solution (Braun). IMA-PVAT was collected immediately after preparation of the arterial bypass graft and before establishing the extracorporeal circulation. C-PVAT was collected during extracorporeal circulation, immediately after removing the aortic cross-clamp, from the same position in all patients, regardless of the localization of atherosclerotic lesions within the coronary tree. PVAT specimens were briefly rinsed with sterile saline and visible connective tissue or blood vessels removed. PVAT samples were then either directly processed under sterile conditions for the preparation of conditioned medium (CM) or stored at −80 °C pending protein or RNA isolation. Specimens intended for RNA isolation were transferred to TRI Reagent® Solution (ThermoFisher Scientific; Dreieich, Germany) before storage at −80 °C. PVAT samples intended for histology/immunohistochemistry were fixed in 10% zinc formalin (Sigma-Aldrich; Darmstadt, Germany), embedded in paraffin wax (Leica; Wetzlar, Germany) and cut into 5-μm-thick sections.

### 2.3. Generation of Perivascular Adipose Tissue Conditioned Medium

For the generation of PVAT-derived CM, C-PVAT and IMA-PVAT specimens were cut into pieces under sterile conditions and then transferred into Dulbecco’s Modified Eagle’s medium (ThermoFisher Scientific) supplemented with 1% penicillin/streptomycin and 0.5% fetal bovine serum (both Biosera). A standard culture medium volume/tissue mass proportion of 2 μL per mg of tissue was employed. Culture medium and PVAT were then incubated in a cell culture incubator (New Brunswick, Eppendorf; Wesseling-Berzdorf, Germany; 37 °C, 5% CO_2_) for 24 h. Control medium was prepared in parallel by incubating medium without PVAT tissue. After removal of PVAT pieces, CM was centrifuged at 20,000 *g* for 10 min and the supernatant kept at −80°C pending analysis.

### 2.4. Membrane-Based Protein Array

C-PVAT- and IMA-PVAT-derived CM (1 mL) from five patients with CAD was examined for secreted proteins using a customized RayBio® membrane-based antibody array against 29 target proteins (RayBiotech, Hölzel Diagnostika Handels GmbH; Köln, Germany), following the instructions of the manual. Chemiluminescence detection was performed using a ChemiDoc™ MP imaging system (Bio-Rad; Rüdigheim, Germany). Semi-quantitative analysis was performed by densitrometry using ImageJ software (version 1.52, NIH) and Gilles Carpentier’s Protein Array Analyser for ImageJ macro (rsb.info.nih.gov/ij/macros/toolsets/Protein Array Analyzer.txt). Negative control dots were used for background subtraction and positive control dots to normalize values and to allow comparison among array membranes. Quantitative data were expressed as fold change vs. IMA-PVAT.

### 2.5. Reverse Transcription Quantitative Real-time PCR

PVAT was homogenized in TRI Reagent® solution using a rotating homogenizer (ART Prozess and Labortechnik GmbH; Müllheim, Germany). For RNA isolation from human dermal lymphatic endothelial cells (HDLECs), cells were directly harvested from the culture plate using TRI Reagent® solution and a cell scraper. RNA isolation was performed using a standard protocol. For PVAT samples, an additional purification step was performed using NucleoSpin® RNA columns (Macherey-Nagel; Düren, Germany). The amount of RNA was quantified on a NanoDrop™ 2000 spectrophotometer (ThermoFisher Scientific). Reverse transcription was performed using M-MLV reverse transcriptase (Promega; Mannheim, Germany) or iScript™ cDNA Synthesis Kit (Bio-Rad). For quantitative real-time PCR (qPCR), SsoAdvanced™ Universal SYBR® Green Supermix (Bio-Rad) and a CFX Connect™ Real-Time PCR Detection System (Bio-Rad) were used. Two technical replicates were prepared for each sample. For PVAT gene expression analysis, LRP10 was used as endogenous reference gene [20]. For HDLECs, preliminary analysis of common reference genes indicated 18S as the most stably expressed gene. The sequences of all primers that were used are shown in Table S1. Data were normalized to the respective reference gene and are expressed as 2^(-DCq)^ values for C-PVAT and IMA-PVAT or as 2^(-DDCq)^ (fold change vs. control-treated samples) for HDLECs.

### 2.6. Spheroid Lymphangiogenesis Assay

HDLECs were cultured in Endothelial Cell Growth Medium MV2 (PromoCell; Heidelberg, Germany) and analyzed up to passage 5. Spheroids of HDLECs were prepared on day one following a standard protocol. In short, cultured HDLECs were detached using 0.05% trypsin-EDTA (ThermoFisher Scientific) and resuspended at a density of 400 cells per 100 µL Endothelial Cell Growth Medium MV2 containing 20% methylcellulose in Medium 199 (both Sigma-Aldrich). Subsequently, 100 μL of the cell suspension were transferred to the wells of a round-bottom 96-well suspension culture plate and incubated for 24 h at 5% CO_2_, 37 °C in order for HDLEC spheroids to form. On day two, spheroids were collected from the 96-well plate. After centrifugation at 390 *g* for three minutes, the supernatant was discarded and spheroids resuspended at a 1:1 mixture of 50% rat tail collagen I (Corning; Wiesbaden, Germany), 0.05% acetic acid (in Medium 199) and 5% fetal bovine serum in methylcellulose/Medium 199 solution. The suspension was transferred to the wells of a 24-well plate and incubated for 30 min before adding Endothelial Cell Growth Medium MV2. Spheroids were stimulated with recombinant human angiopoietin-2 (ANGPT2; 100 ng/mL) and/or recombinant human vascular endothelial growth factor (VEGF)-C (100 ng/mL; both from R&D Systems) for 24 h. Images of five randomly selected spheroids per well were captured using an inverted microscope (Motic AE31; Wetzlar, Germany). The number of sprouts was manually counted and the cumulative sprout length measured using ImageJ software.

### 2.7. Histology and Immunohistochemistry

Masson’s trichrome stain was used for the histological examination of PVAT specimens. For the immunohistochemical staining of the paraffin-embedded tissue sections, a standardized protocol was used. Briefly, paraffin sections were deparaffinized and epitopes were retrieved by heating in 10 mM Tris buffer containing 1 mM EDTA or in 0.1 M citrate buffer (pH 6.0), depending on the target epitope. Unspecific binding sites were blocked using 10% normal goat serum (Abcam; Cambridge, UK). Vessel endothelium was stained using an anti-PECAM-1 antibody (mouse monoclonal; Dako; Santa Clara, CA, USA). Anti-podoplanin (PDPN; rabbit polyclonal; Novus Biologicals) and anti-lymphatic vessel endothelial hyaluronan receptor 1 (LYVE-1; rabbit polyclonal; Novus Biologicals) antibodies were used to selectively stain lymphatic endothelium. Tissue macrophages were visualized using anti-CD68 antibodies (mouse monoclonal; Dako), ANGPT2-expressing cells using anti-ANGPT2 antibody (rabbit polyclonal; Novus Biologicals; Wiesbaden, Germany). After incubating with biotinylated secondary goat anti-mouse or anti-rabbit antibodies (ThermoFisher Scientific), sections were incubated with avidin–biotin complex (Vector Laboratories; Burlingame, CA, USA). Diaminobenzidine or aminoethylcarbazole (Vector Laboratories) substrate was used for visualization, followed by brief counterstaining using Gill’s hematoxyline (Sigma-Aldrich). Negative controls were prepared for all immunohistochemical stainings by omitting the primary antibody (representative images shown in Figure S1).

### 2.8. Image Acquisition and Analysis

Histology and immunohistochemistry sections were examined and representative images were captured using an Olympus BX51 microscope (Hamburg, Germany). The number of total vessels and lymphatic collector vessels was quantified by manually counting PECAM-1- and PDPN-immunopositive vessel-like structures per 200× microscope field, respectively. The number of lymphatic capillaries was quantified by manually counting LYVE-1-immunopositive structures per 200× microscope field. The number of tissue macrophages was determined by manually counting CD68-immunopositive cells per 400× microscope field. Results were expressed as number of immunopositive vessel-like structures or cells per mm^2^. Sections stained with Masson’s trichrome stain were used for the assessment of fibrosis, which was performed by quantifying the blue-stained collagen area relative to total tissue area at 100× magnification. For all quantitative image analyses, three optical fields were randomly selected and measurements averaged.

### 2.9. Statistical Analysis

Data are presented as mean ± standard deviation (SD), if normally distributed, or as median (25% and 75% interquartile range (IQR)), if not. Normal distribution was examined with the D’Agostino–Pearson omnibus normality test. Paired comparison analyses between PVAT samples from one patient were performed using Student’s paired *t*-test if values were normally distributed, or the Wilcoxon matched-pairs signed rank test if not. Multiple comparisons were performed using one-way ANOVA with Turkey’s multiple comparisons test. Differences were considered statistically significant if *p* < 0.05. All statistical analyses were performed using GraphPad PRISM version 8.0.

## 3. Results

### 3.1. C-PVAT Contains More Lymphatic Vessels than IMA-PVAT

**Figure 1:**
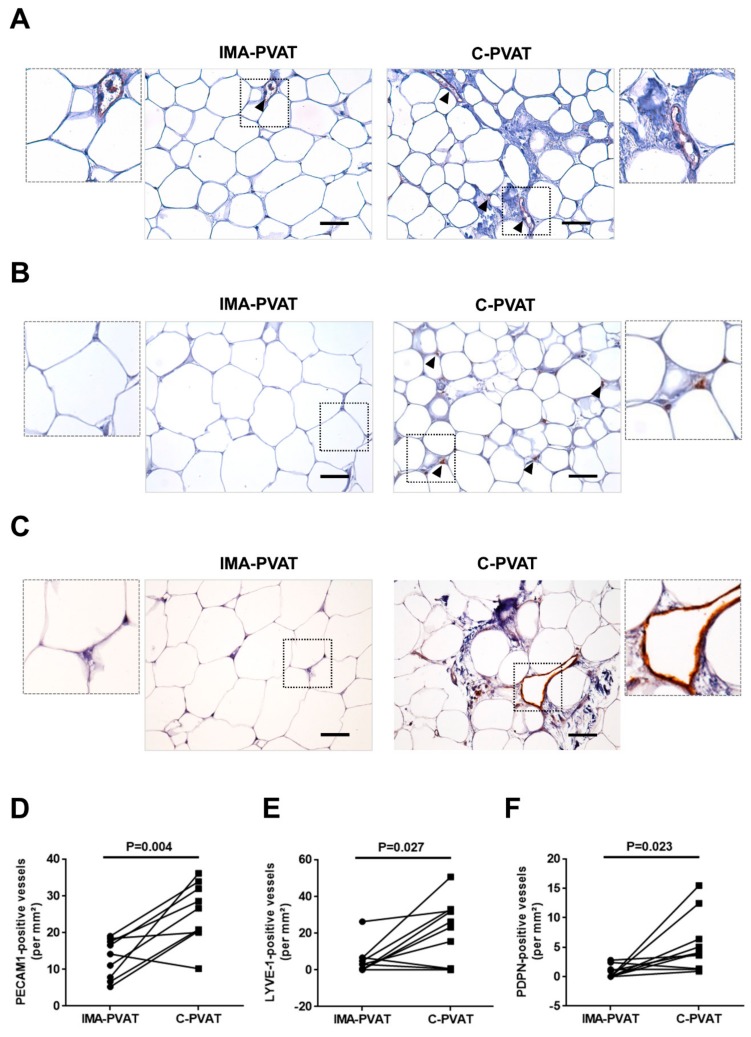
Angiogenesis and lymphangiogenesis in perivascular adipose tissue (PVAT). (**A–C**) Immunohistochemical detection of platelet endothelial cell adhesion molecule 1 (PECAM-1) (**A**), anti-lymphatic vessel endothelial hyaluronan receptor 1 (LYVE-1) (**B**), and anti-podoplanin (PDPN) (**C**) in internal mammary artery (IMA)-PVAT and cardiac (C)-PVAT. Inserts show higher magnification. Size bars represent 100 μm. (**D**–**F**) Quantification of the number of PECAM-1 (**D**), LYVE-1 (**E**), and PDPN (**F**) immunopositive vessels per mm^2^ in *n* = 10 patients with coronary artery disease (CAD). Paired IMA-PVAT (●) and C-PVAT (■) values in individual patients are connected with a line. Statistical analysis was performed using Student’s paired *t*-test (**D**,**F**) or Wilcoxon matched-pairs signed rank test (**E**).

We had previously shown that C-PVAT from patients with CAD is characterized by more pronounced angiogenesis and higher numbers of blood vessels compared to IMA-PVAT [20]. Immunohistochemical detection of PECAM-1 in paired PVAT tissue specimens from 10 patients with CAD confirmed those previous findings by showing a significantly higher density of PECAM-1-immunopositive vessels in C-PVAT compared to IMA-PVAT (25.4 ± 2.75 vs. 12.9 ± 1.79 per mm^2^, *p* = 0.004; Figure 1A,D; for findings in negative controls please see Figure S1A). To distinguish lymphatic vessels, we used markers expressed on lymphatic endothelium, namely LYVE-1 and PDPN. LYVE-1 has been shown to be mainly expressed in lymphatic capillaries, PDPN in lymphatic collector vessels [23]. These analyses revealed that both the number of LYVE-1 (24.7 (0.61–32.3) vs. 2.97 (0–6.58) per mm^2^, *p* = 0.027; Figure 1B,E; Figure S1B) and PDPN (5.45 ± 1.54 vs. 0.74 ± 0.34 per mm^2^, *p* = 0.023; Figure 1C,F; Figure S1C) -immunopositive vessels was significantly increased in C-PVAT compared to IMA-PVAT. Of note, neither the anti-LYVE-1 nor the anti-PDPN antibody showed any immunoreactivity for endothelium lining blood vessels. Interestingly, the mean diameter of PDPN-positive vessels was significantly increased in C-PVAT compared to IMA-PVAT (*p* = 0.008), in line with lymphatic dysfunction [14,15].

### 3.2. Lymphangiogenic Growth Factors and Their Receptors Are Expressed at Higher Levels in C-PVAT Compared to IMA-PVAT

**Figure 2:**
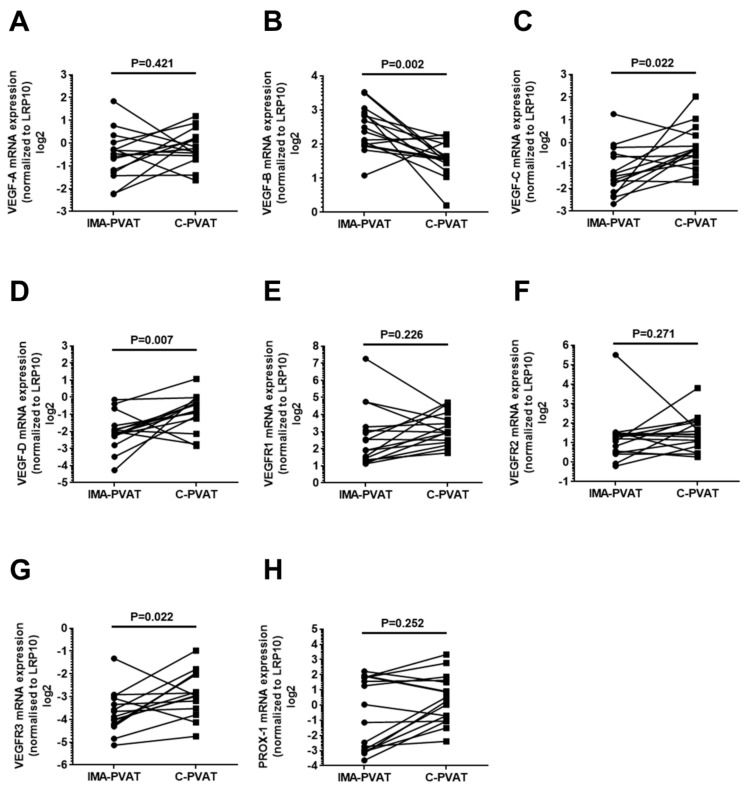
Analysis of growth factors involved in angiogenesis and lymphangiogenesis. Quantification of mRNA expression of vascular endothelial growth factor (*VEGF*)*-A* (**A**), *VEGF-B* (**B**), *VEGF-C* (**C**), *VEGF-D* (**D**), VEGF receptor (*VEGFR*)*-1* (**E**), *VEGFR2* (**F**), *VEGFR3* (**G**), and prospero homeobox-1 (*PROX-1*) (**H**) in IMA-PVAT and C-PVAT of *n* = 15–16 patients with CAD. Paired IMA-PVAT (●) and C-PVAT (■) values in individual patients are connected with a line. Statistical analysis was performed using Wilcoxon matched-pairs signed rank test.

To examine whether the more extensive lymphatic network in C-PVAT is accompanied by a higher expression of growth factors known to mediate (lymph-) angiogenesis, qPCR analysis was employed to determine gene expression levels of vascular endothelial growth factor (VEGF)-A, VEGF-B, VEGF-C, and VEGF-D in C-PVAT and IMA-PVAT (paired tissue samples from 16–18 patients). These analyses revealed no significant differences in the mRNA levels of *VEGF-A* (0.85 (0.68–1.21) vs. 0.72 (0.41–1.02), *p* = 0.421; Figure 2A), whereas the expression of *VEGF-C* (0.83 (0.55–1.25) vs. 0.36 (0.22–0.72), *p* = 0.022; Figure 2C) and *VEGF-D* (0.58 (0.42–0.87) vs. 0.25 (0.21–0.31), *p* = 0.007; Figure 2D) was significantly increased in C-PVAT compared to IMA-PVAT. On the other hand, *VEGF-B* mRNA levels were significantly reduced in C-PVAT compared to IMA-PVAT (5.38 (3.92–7.53) vs. 2.99 (2.72–4.13), *p* = 0.002; Figure 2B).

We then also examined the mRNA expression of the receptors for VEGF, namely VEGF receptor (VEGFR)-1, VEGFR2, and VEGFR3. In agreement with our findings of a significantly higher VEGF-C and VEGF-D expression in C-PVAT, mRNA levels of *VEGFR3* were significantly increased in C-PVAT compared to IMA-PVAT (0.13 (0.09–0.25) vs. 0.07 (0.05–0.12), *p* = 0.022; Figure 2G), whereas no differences in the mRNA expression of *VEGFR1* (7.78 (5.29–16.2) vs. 4.70 (2.59–9.37), *p* = 0.226; Figure 2E) and *VEGFR2* (2.68 (1.85–4.15) vs. 2.38 (1.41–2.79), *p* = 0.271; Figure 2F) were observed between both PVAT depots. These results suggested a higher state of VEGF-C/VEGF-D-mediated VEGFR3 signaling which may be responsible for the observed differences in the lymphatic endothelial cell density between C-PVAT and IMA-PVAT.

Analysis of the transcription factor prospero-related homeobox (PROX)-1, a master regulator of embryonic lymphatic development [24,25], revealed similar *PROX-1* mRNA levels in C-PVAT and IMA-PVAT (1.31 (0.51–3.01) vs. 0.74 (0.12–3.50), *p* = 0.252; Figure 2H) suggesting that the observed differences in the number of lymphatic vessels was not the result of a different embryonically defined lymphangiogenic potential in both adipose tissue depots.

### 3.3. Increased Inflammation and Fibrosis in PVAT Surrounding Atherosclerotic Arteries

**Figure 3:**
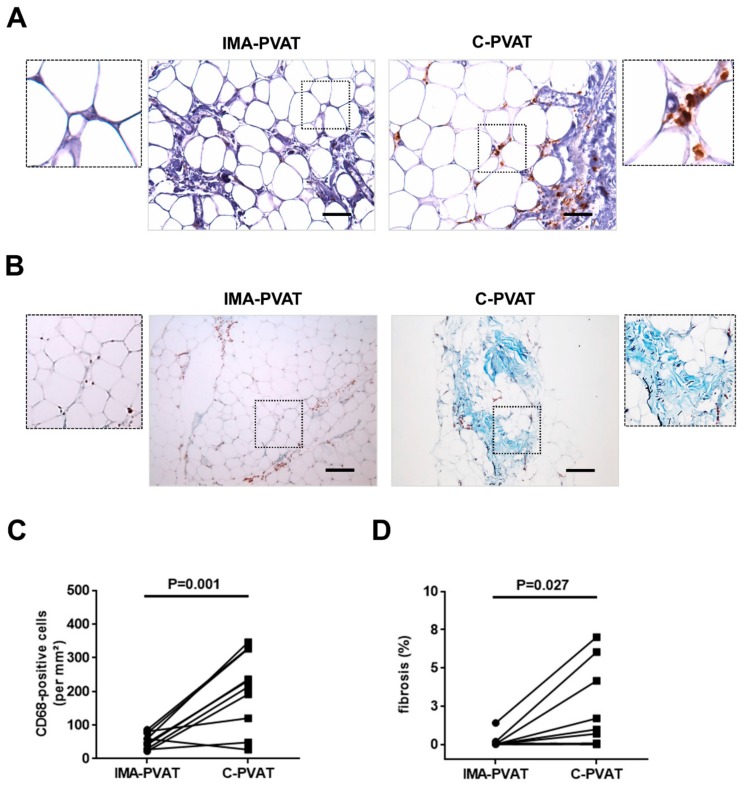
Fibrosis and inflammation in PVAT. (**A**) Immunohistochemical detection of CD68 in IMA-PVAT and C-PVAT. Size bars represent 50 μm. (**B**) Masson trichrome stain in IMA-PVAT and C-PVAT. Size bars represent 200 μm. (**C**) Quantification of the number of CD68-immunopositive cells per mm^2^. (**D**) Quantification of the percentage of fibrotic area in *n* = 10 patients with coronary artery disease (CAD). Paired IMA-PVAT (●) and C-PVAT (■) values in individual patients are connected with a line. Statistical analysis was performed using Student’s paired *t*-test (**C**) or Wilcoxon matched-pairs signed rank test (**D**).

Previous studies have shown that inflammation triggers lymphangiogenesis and identified macrophages as main cellular source of VEGF-C and VEGF-D [26,27]. Higher numbers of CD68-immunopositive macrophages were observed in human C-PVAT compared to IMA-PVAT of patients with CAD (207 ± 35.8 vs. 52.9 ± 7.32 per mm^2^, *p* = 0.001; Figure 3A,C; Figure S1D). Because of the complex interplay between lymphangiogenesis, inflammation and fibrosis, we next examined both PVAT depots after staining with Masson’s trichrome stain. These analyses revealed a significantly higher degree of fibrosis in C-PVAT compared to IMA-PVAT (0.83 (0.02–4.63) vs. 0.02 (0.01–0.11) % per microscope field, *p* = 0.027; Figure 3B,D). Of note, real time PCR analysis did not reveal differences of transforming growth factor-beta (*TGFβ*) mRNA levels between both adipose tissue depots (*p* = 0.151).

### 3.4. Protein Array Analysis Reveals Significantly Higher Angiopoietin-2 and Intercellular Adhesion Molecule-1 Levels in C-PVAT Compared to IMA-PVAT

**Figure 4:**
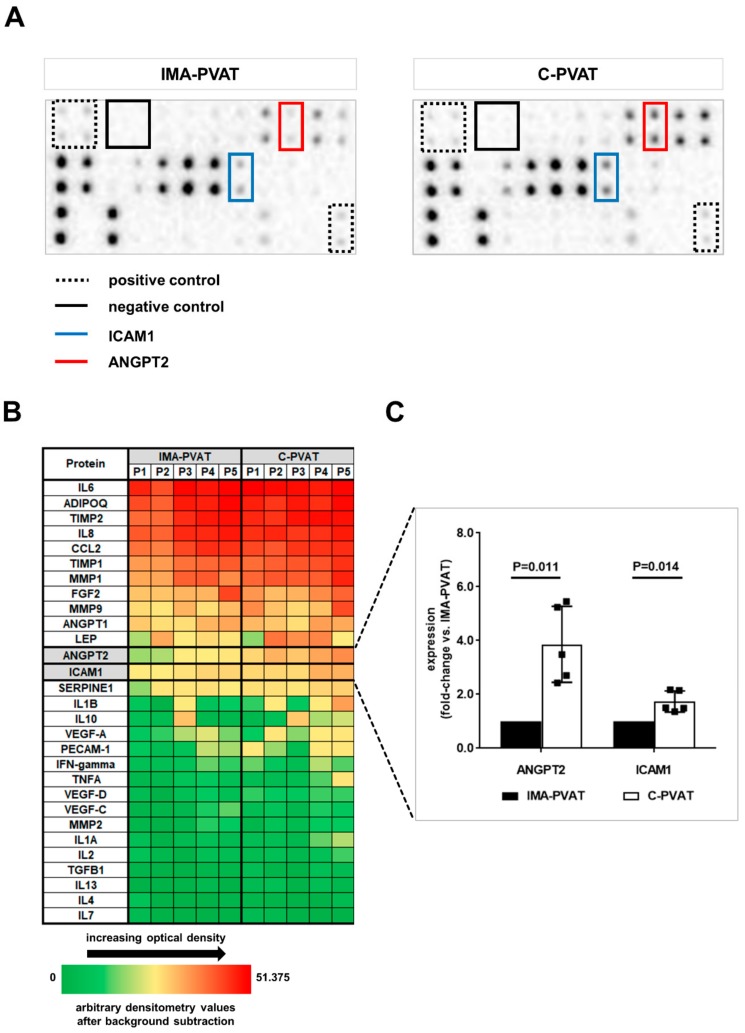
Protein array analysis of factors potentially involved in perivascular lymphangiogenesis. (**A**) Representative chemiluminescence detection images of a protein membrane array in IMA-PVAT- and C-PVAT-derived conditioned medium from one patient with CAD. A commercially available membrane-based antibody protein array was used. Each vertical pair of dots represents one protein. Boxes of the same color on both membranes mark the respective dot pairs for each protein. Positive and negative control dots are also marked. (**B**) Heat map representation of the expression of 29 proteins quantified using membrane-based protein array analysis in IMA-PVAT- and C-PVAT-derived conditioned medium of *n* = 5 patients with CAD. Each column represents one patient (P1, P2, etc.). (**C**) Quantitative analysis shown for proteins exhibiting a statistically significant difference in expression between IMA-PVAT and C-PVAT. Bars represent fold change of mean protein expression compared to IMA-PVAT. Error bars represent standard deviation. Statistical analysis was performed using Student’s paired *t*-test.

PVAT has been shown to express and secrete a number of cytokines and other factors, which may act locally or in a paracrine manner on the neighboring vessel wall (reviewed in [28]). For this reason and given our findings, we next aimed at investigating a broader spectrum of PVAT-secreted factors, with particular focus on potential candidates involved in (lymph)angiogenesis, inflammation and fibrosis. A semiquantitative paired expression analysis of 29 target proteins was performed in conditioned medium (CM) derived from C-PVAT and IMA-PVAT of five patients with CAD using a membrane-based antibody array (representative results are shown in Figure 4A, the position of all target proteins on the membranes is given in Figure S2). Among those, inflammatory factors (interleukin-6, interleukin-8, monocyte chemoattractant protein-1), matrix metalloproteinases (MMP1, MMP9) and their inhibitors (tissue inhibitor of metalloproteinases TIMP1, TIMP2), adipokines (adiponectin, leptin) and growth factors (angiopoietin-1 and -2, basic fibroblast growth factor) were detected in CM from both adipose tissue depots (heat map in Figure 4B, quantitative analyses for all 29 target proteins in Figure S3A–D).

**Figure 5:**
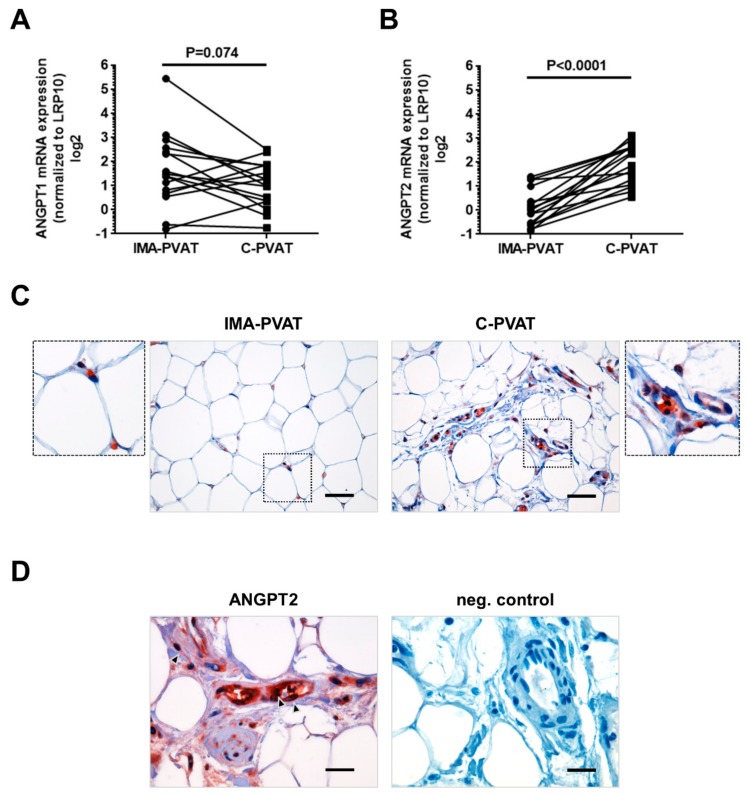
Expression of angiopoietin-1 and -2 in PVAT. (**A**) Quantification of mRNA expression of angiopoietin-1 (ANGPT1) (**A**) and angiopoietin-2, ANGPT2 (**B**) in IMA-PVAT and C-PVAT of *n* = 16 patients with CAD. Paired IMA-PVAT (●) and C-PVAT (■) values of individual patient are connected with a line. (**C**) Representative image after immunohistochemical detection of ANGPT2 in IMA-PVAT and C-PVAT. Size bars represent 50 μm. (**D**) Higher magnification showing ANGPT2-positive cells (arrows). Negative control after omitting the primary antibody is also shown. Size bars represent 20 μm. Statistical analysis was performed using Wilcoxon matched-pairs signed rank test.

Among the most abundant factors, ANGPT2 (*p* = 0.011) and ICAM1 (*p* = 0.014) protein levels were found to significantly differ, with higher levels in C-PVAT compared to IMA-PVAT (Figure 4B). ANGPT2 overexpression in C-PVAT could be confirmed on the transcriptional level by qPCR analysis in 16 patients with CAD, showing significantly higher *ANGPT2* mRNA levels in C-PVAT compared to IMA-PVAT (1.31 (0.51–3.01) vs. 0.74 (0.12–3.50), *p* < 0.0001; Figure 5B). On the other hand, *ANGPT1* mRNA levels did not significantly differ between C-PVAT and IMA-PVAT (2.18 (1.28–5.73) vs. 2.70 (1.63–5.73), *p* = 0.074; Figure 5A). Immunohistochemical staining of ANGPT2 identified endothelium, but also infiltrating immune cells and fibroblasts as possible cellular source of ANGPT2 in PVAT specimens (Figure 5D), both of which were more abundant in C-PVAT (Figure 5C). These findings suggested that the increased expression of ANGPT2 in C-PVAT compared to IMA-PVAT represents the composite result of increased vascularization, inflammation, and fibrosis. Protein levels of ICAM1 were significantly elevated in C-PVAT compared to IMA-PVAT (Figure 4B), in line with the presence of inflamed lymphatic endothelium [29]. Moreover, VEGF-D levels were also significantly increased in C-PVAT compared to IMA-PVAT, in accordance with the difference at mRNA level, whereas no statistically significant increase was detected for VEGF-C (Figure S3A). The expression of MMP9 was also found to be increased in C-PVAT compared to IMA-PVAT, the difference approaching but not reaching statistical significance (*p* = 0.057).

### 3.5. Recombinant ANGPT2 Stimulates Sprouting Angiogenesis of Human Lymphatic Endothelial Cells

To further study the importance of the above observations for perivascular adipose tissue lymphangiogenesis, the effect of recombinant human ANGPT2 was examined employing the three-dimensional spheroid angiogenesis assay in human lymphatic endothelial cells (HDLECs). Of note, total CM from C-PVAT and IMA-PVAT did not differ in its ability to promote HDLECs angiogenesis in the matrigel™ (Figure S4A–C) or the spheroid assay (Figure S4D–F); however, due to the fact that PVAT-derived conditioned medium contains a mixture of numerous factors, the effects of one specific factor on cultivated cells in vitro may have been obscured.

**Figure 6:**
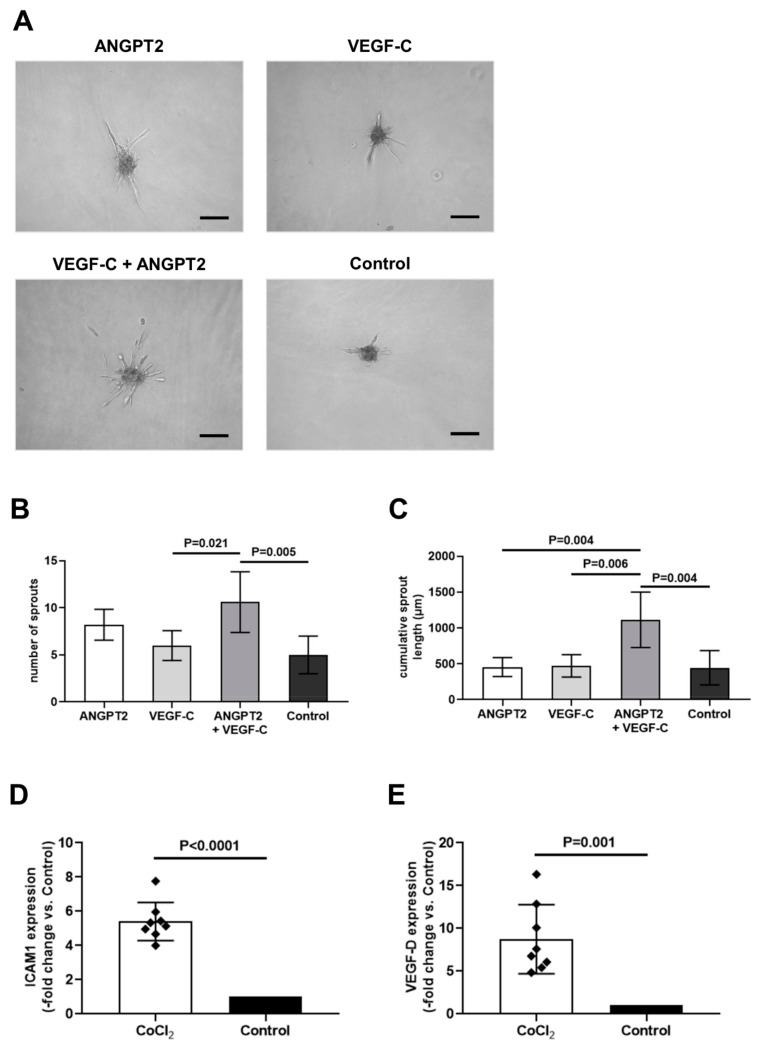
Effects of ANGPT2 and VEGF-C on human sprouting lymphangiogenesis. Representative images of human dermal lymphatic endothelial cell (HDLEC) spheroids (**A**) as well as the results after quantitative analysis of the number of sprouts (per spheroid) (**B**) and the cumulative sprout length (µm) in *n* = 5 spheroids (**C**) after stimulation with recombinant human ANGPT2; (100 ng/mL) and/or VEGF-C (100 ng/mL) or control (CTL). Size bars represent 200 μm. (**D–E**) Quantification of mRNA expression of ICAM1 (**D**) and VEGF-D (**E**) in HDLECs, after induction of chemical hypoxia using 1 mM of CoCl_2_ for 4 h in *n* = 8 independent experiments. Bars represent fold change of mean mRNA expression compared to Control. Error bars represent standard deviation. Statistical analysis was performed using one-way ANOVA with Turkey’s multiple comparisons test (**B**,**C**) and Student’s paired *t*-test (**D**,**E**).

Flow cytometry analysis confirmed that HDLECs express (lymphatic) endothelial cell markers, including VEGFR3, the receptor for VEGF-C, and TIE2 (TEK receptor tyrosine kinase), the receptor for ANGPT2 (Figure S5). Stimulation of HDLEC spheroids with ANGPT2 (100 ng/mL) for 24 h did not alter the number of sprouts (Figure 6A,B) or the cumulative sprout length (Figure 6A,C) compared to control-treated spheroids. Stimulation of HDLECs spheroids with ANGPT2 together with VEGF-C (100 ng/mL each), both overexpressed (ANGPT2 on the mRNA and the protein level, VEGF-C on the mRNA level) in C-PVAT, significantly increased both the number of sprouts (*p* = 0.005 vs. control, *p* = 0.021 vs. VEGF-C; Figure 6B) and the cumulative sprout length (*p* = 0.004 vs. control and ANGPT2, *p* = 0.006 vs. VEGF-C; Figure 6C). These findings are in agreement with the more extended lymphatic network in C-PVAT compared to IMA-PVAT observed in this study and previous findings on the role of ANGPT2 and VEGF-C as important regulators of functional lymphatic vessel formation [30,31,32].

Previous studies have shown that inflammatory cytokines, in particular TNFα, is capable of inducing ANPGT2, but also ICAM1 expression suggesting a role of ANGPT2 in inflammatory lymphangiogenesis [33]. Because TNFα levels did not differ in CM from either of the PVAT depots (Figure S3C), we examined the possible role of perivascular hypoxia in the observed overexpression of ANGPT2 and ICAM1. Chemical hypoxia was induced on cultured HDLECs by stimulation with 1 mM cobalt chloride (CoCl_2_) for 4 h (eight independent experiments). The mRNA expression of ICAM1 was found to be significantly increased in CoCl_2_-treated compared to control-treated HDLECs (5.4 fold increase vs. control; *p* < 0.0001; Figure 6D). VEGF-D mRNA levels were also significantly increased (8.7 fold increase vs. control; *p* = 0.001; Figure 6E), whereas no significant differences were found in the mRNA expression of ANGPT2, VEGF-C, TIE2 and VEGFR3.

## 4. Discussion

The main findings of our study are that (1) PVAT surrounding the aortic root and the coronary arteries of patients with CAD exhibits a denser lymphatic vessel network compared to PVAT surrounding the IMA, an ‘atherosclerosis-resistant’ artery; (2) The expression of lymphangiogenic growth factors (VEGF-C and VEGF-D) and their primary receptor (VEGFR3) is significantly increased in C-PVAT compared to IMA-PVAT, both at the mRNA (VEGF-C and VEGF-D) and the protein level (VEGF-D); (3) Increased lymphangiogenesis was accompanied with increased expression of ICAM1, infiltration with macrophages and more extensive fibrosis; and (4) Elevated levels of ANGPT2 in C-PVAT could be identified as one potential factor contributing to the observed increase in perivascular lymphangiogenesis. To the best of our knowledge, the present study is the first to compare lymphangiogenesis and its regulators in the perivascular fat surrounding an atherosclerotic and a non-diseased vessel in patients with coronary artery disease. Regarding the use of the IMA as ‘control’ artery it should be noted that, although the reason for the observed resistance of this artery to atherosclerosis is unclear [34], PVAT surrounding the IMA has been reported to exert vasodilatatory effects on the underlying vessel [35], which supports the hypothesis that IMA-PVAT may in part be responsible for the protection of this artery against atherosclerosis. Previous findings of marked gene expression heterogeneity between PVAT surrounding the coronary artery and the IMA in patients with CAD also support the contribution of differences in perivascular fat composition to the susceptibility to atherosclerosis [36,37]. On the other hand, C-PVAT from healthy individuals cannot be surgically obtained and is therefore not available as ‘control’ PVAT depot in patients with CAD undergoing bypass surgery.

The possible role of the adventitia and perivascular adipose tissue in the pathophysiology of atherosclerosis has only recently received attention. Several studies, including from our own group, identified factors expressed and released in the perivascular fat as important local mediators of vascular repair processes and atherosclerotic lesion formation (reviewed in [28]). Some of the factors overexpressed in PVAT possess angiogenic activities, such as leptin [38] or CCL2 [39], and adventitial neoangiogenesis may contribute to atherosclerotic plaque progression and instability [1,2,3,4,5,6]. In contrast to perivascular networks created by blood endothelial cells, studies on the possible role of adventitial lymphangiogenesis in atherosclerosis are limited. For example, dissection of the plaque draining lymph node in apolipoprotein E-knockout mice aggravated atherosclerotic burden [11], whereas restoration of lymphatic drainage in hypercholesterolemic mice improved reverse cholesterol transport. On the other hand, and to the best of our knowledge, animal studies have so far not addressed the importance of coronary perivascular lymphangiogenesis during atherosclerosis. On the other hand, post-mortem analyses in humans and a clinical study found a more extended lymphatic vasculature in the adventitia of atherosclerotic vessels [11,16,17,18,19]. Although contradictory at first, both observations may reflect different stages of a chronic disease process in which perivascular lymph vessels reactively develop to accelerate the removal of lipids and the exit of inflammatory cells, but become exhausted and dysfunctional at later stages due to chronic overactivation [8,15]. Although our study cannot provide a definite answer to the important question of whether the observed increased lymphangiogenesis in C-PVAT is the consequence of the existing atherosclerosis in human coronary arteries or one of its causes, the increased expression of ICAM1, infiltration with macrophages, and fibrosis observed in C-PVAT appear to be in accordance with the concept of reactive, i.e. inflammation- or fibrosis-induced, adventitial lymphangiogenesis.

**Figure 7:**
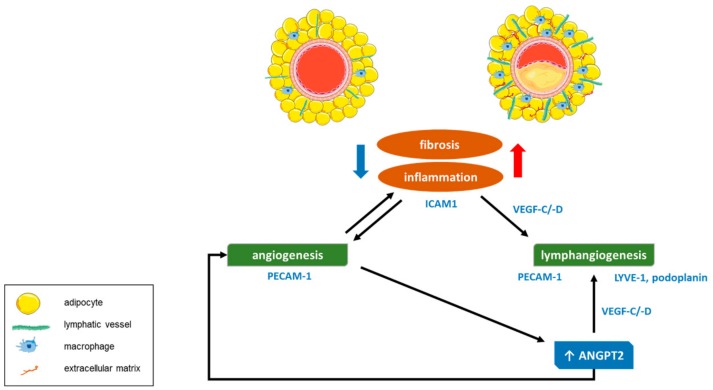
Schematic drawing depicting the main findings of this study.

Overall, the findings of this and previous studies emphasize that the connections among (peri)vascular inflammation, fibrosis and (lymph)angiogenesis are rather complex and that the pathophysiological pathways underlying our observations are probably not unidirectional. Available data rather propose a circular mechanism with multiple shortcuts among various biological processes (schematically depicted in Figure 7), in which advanced atherosclerosis with arterial wall inflammation and fibrosis promote perivascular angiogenesis and lymphangiogenesis.

Regarding the signals mediating perivascular lymphangiogenesis, the increase in lymphatic vessels in C-PVAT compared to IMA-PVAT was accompanied with elevated mRNA levels of two major regulators of lymphangiogenesis, namely VEGF-C and VEGF-D, as well as their receptor VEGFR3. Increased numbers of intimal cells expressing VEGF-C were also reported in patients with atherosclerosis of the iliac arteries and found to correlate with atherosclerotic lesion severity and the number of LYVE1-positive lymphatic vessels, whereas similar associations were not observed for VEGF-D [18]. Macrophages are an important source of VEGF-C and VEGF-D, as shown in tumors [26,27,40], and both growth factors may also act as chemoattractant by upregulating the expression of VEGFR3 on M1 polarized macrophages, as shown in visceral adipose tissue of mice [41]. TGFβ [42,43] and proinflammatory cytokines, such as interleukin (IL) 1β and TNFα [44], have been shown to further enhance the expression of VEGF-C and to promote lymphangiogenesis, although we did not detect differences in their expression levels between both adipose tissue depots.

The importance of VEGF-C−VEGFR3 in the regulation of lymphangiogenesis is well established [31,45,46]. However, under some circumstances VEGF-C may also bind to VEGFR2, expressed on lymphatic and blood endothelial cells [47,48]. VEGF-C was shown to promote angiogenesis in the developing embryo [49] or following ischemia [50]. Others found that VEGF-C overexpression selectively induced lymphangiogenesis without altering accompanying angiogenesis [45,51]. The net result of VEGF-C signaling in terms of (lymph)angiogenesis may depend on the local abundance of VEGFR2 and VEGFR3 receptors, as findings in mice expressing a VEGFR3-specific mutant of VEGF-C exhibited growth of lymphatic, but not of blood vessels [52]. VEGF-D also binds both VEGFR2 and VEGFR3 with high affinity [53] and has angiogenic as well as lymphangiogenic activities [54,55]. On the other hand, the expression of VEGF-A, an essential hemangiogenic factor, did not significantly differ between C-PVAT and IMA-PVAT, whereas VEGF-B, a specific ligand for VEGFR1 with limited angiogenic potential, was markedly reduced in our study. Although VEGF-B is dispensable for blood vessel growth, it was found to have pro-survival effects on endothelial cells, vascular smooth muscle cells and pericytes [56]. Interestingly, VEGF-B is highly expressed in brown adipose tissue [57] and was shown to control endothelial fatty acid uptake [58]. The significantly reduced expression observed in C-PVAT may reflect the loss of protective (brown) adipose tissue properties compared to IMA-PVAT, as suggested by findings in rodents [59].

ANGPT2 holds a significant role in postnatal angiogenic remodeling, but also the proper development of lymphatic vessels [30,60]. Here, we show that the expression of ANGPT2 is increased in C-PVAT compared to IMA-PVAT in patients with CAD, and findings could be confirmed at the protein level in the secretome of C-PVAT and IMA-PVAT. Others have shown that ANGPT2 overexpression leads to lymphatic hyperplasia [30], suggesting that increased ANGPT2 levels may have contributed to the enhanced lymphangiogenic vessel density observed in this PVAT depot. In agreement with the described synergistic effect of VEGF and ANGPT2 in promoting lymphangiogenesis [60,61], we could show that stimulation of HDLEC spheroids using a combination of ANGPT2 and VEGF-C results in lymphangiogenic sprouting, whereas stimulation with ANPGT2 or VEGF-C alone had weak or no effects at all. Although ANGPT2 is mainly expressed in endothelial cells [62], immunohistochemical analysis revealed ANGPT2 protein expression also in infiltrating immune cells and myofibroblasts, in line with their role as important source of lymphangiogenic growth factors. Binding of ANGPT2 to TIE2 receptors may activate NFκB (nuclear factor ‘kappa-light-chain-enhancer’ of activated B-cells) signaling and the expression of adhesion receptors on endothelial cells, including ICAM1 [63]. NFkB may also upregulate the expression of VEGFR3 on lymphatic endothelial cells and increase their sensitivity to VEGF-C and -D [64], and analyses in mice or human endothelial cells lacking ANGPT2 showed that it may increase the responsiveness of vascular endothelial cells to inflammatory stimuli [65].

In addition to inflammatory signals, hypoxia may be a relevant factor underlying the observed elevated lymphangiogenic growth factor levels in C-PVAT. Hypoxic cells, such as those present in tumors or atherosclerotic plaques, may release angiogenic growth factors, and we have previously shown that hypoxia levels are higher in C-PVAT compared to IMA-PVAT and that this difference may underlie the increased expression of leptin, an adipokine with proangiogenic properties, in the C-PVAT of CAD patients [20]. Hypoxia is also a major stimulus inducing the expression of VEGF. In the present study, inhibition of hypoxia-inducible factor 1-alpha (HIF1α) degradation by CoCl_2_ did not alter the expression of ANGPT2 and VEGF-C or their receptors, but significantly increased the expression of VEGF-D and ICAM-1. A previous study showed that the VEGF-C promotor does not contain hypoxia-responsive elements, and that hypoxia may induces VEGF-C via HIF1α-independent mechanisms [66]. Interestingly, lymphatic vessels respond to the same angiogenic cues as endothelial cells lining blood vessels, although they exhibit unidirectional flow and do not transport erythrocytes and thus will not improve oxygen supply to hypoxic tissues. The parallel increase of blood and lymph vessel formation could represent a mechanism to remove excess interstitial fluid and cells extravasated from immature and leaky angiogenic blood vessels during hypoxia.

Our study has some limitations. Due to the small amount of tissue available from each patient, the total study collective had to be split into groups for different types of assays, which may have influenced our observations. It should also be noted that ‘only’ PVAT but not specimens from the underlying vascular wall were available for analysis, and that we could not directly associate our findings in PVAT with the severity of the atherosclerotic lesion in the underlying artery. Also, perioperative C-PVAT sampling was performed in a standardized manner (near the aortic root), regardless of the localization of atherosclerotic lesions within the coronary tree. All individuals included in the present study were diagnosed with advanced coronary atherosclerosis with indication for a bypass graft surgery, and our study could therefore not examine earlier stages of this chronic vascular wall disease evolving over many years. Also, C-PVAT from healthy individuals is not available for analysis and thus the distribution of lymphatic vessels in C-PVAT under ‘normal’ conditions is unknown. On the other hand, using the IMA as an internal ‘healthy’ control artery may have helped to provide additional insights into the pathophysiological role of local processes, in particular perivascular lymphangiogenesis during atherosclerosis, whereas the possible impact of systemic effects on the composition and expression patterns should have been minimized by the direct comparison of these two PVAT depots from the same patient. However, it cannot be excluded that differences in the local microenvironment between the two PVAT depots may have contributed to the observed differences.

In conclusion, our findings show an increased lymphangiogenic activity in PVAT surrounding atherosclerotic coronary arteries compared to PVAT surrounding the IMA, an artery free of atherosclerosis, in patients with CAD. Increased ANGPT2 expression and activated VEGF-C/VEGF-D-mediated VEGFR3 signaling, in combination with a local proinflammatory and hypoxic environment, may pathophysiologically underlie these observations.

## Supplementary Materials

The following are available online at https://www.mdpi.com/2077-0383/8/7/1000/s1; Figure S1: Immunohistochemical analysis of PVAT: negative controls; Figure S2: Cytokine antibody array map; Figure S3: Quantitative analysis of protein expression in PVAT using cytokine antibody arrays; Figure S4: Analysis of paracrine effects of PVAT-derived conditioned medium on human dermal lymphatic endothelial cells; Figure S5: Flow cytometry analysis of (lymphatic) endothelial cell markers in HDLECs; Table S1: Primer sequences used for gene expression analysis of human PVAT.
