# Supplementary material for: Increased Lymphangiogenesis and Lymphangiogenic Growth Factor Expression in Perivascular Adipose Tissue of Patients with Coronary Artery Disease
Source: J Clin Med. 2019 Jul 9;8(7):1000. doi: 10.3390/jcm8071000 (PMC6678243; doi:10.3390/jcm8071000)
Supplement: Supplementary file 1 [file jcm-08-01000-s001.pdf]

Supplemental Material

Supplemental Methods

Matrigel-based tube formation assay

The wells of a 24-well suspension culture plate were coated with 280 µL Matrigel® matrix (Corning) and incubated in a standard cell culture incubator for 30 minutes, in order for the matrix to polymerize. Human dermal lymphatic endothelial cells (HDLECs; PromoCell) were conditioned in Endothelial Cell Basal Medium MV2 (EBM; PromoCell) containing 5% fetal bovine serum (FBS; Biosera) for 2 hours. Then, cells were detached using 0.05% Trypsin-EDTA (ThermoFisher Scientific), plated in the Matrigel®-coated 24-well plates at a density of 40,000 cells per well. Next, 450 µL of EBM / 5% FBS was mixed with 50 µl PVAT-derived CM and incubated at 37°C / 5% CO_2 for 24 hours. Pictures were captured from the center of each well using a Motic AE31 inverted microscope. The number of segments and the number of intersections per picture was quantified manually.

Flow cytometry

HDLECs were detached from the culture plate using 0.05% trypsin-EDTA (ThermoFisher Scientific) and fixed using 0.1% paraformaldehyde (Sigma-Aldrich) in phosphate buffer saline (Gibco) for 10 minutes. Following fixation, cells were resuspended in flow cytometry staining buffer (1% fetal bovine serum, 2 mM ethylenediaminetetraacetic acid in phosphate buffer saline) and stored at 4°C pending analysis. On the day of analysis, cells were permeabilized using 0.2% Triton® X 100 (Roth) and incubated with Fc receptor inhibitor antibody (eBioscience) for 20 minutes. Staining was performed in flow cytometry staining buffer using antibodies directly conjugated to a fluorochrome (BioLegend) for 30 minutes. Unstained controls were prepared by omitting antibody. Acquisition was performed using a BD FACSCanto flow cytometer (BD Biosciences) and analysis using FlowJo version 10.

Supplemental Tables

Table 1. Primer sequences used for gene expression analysis of human PVAT.

Factor	Gene	Primer sequences (in 5′–3′ direction)	T_m (°C)	cycles
**18S**	*18S*	F: CGAAAGCATTTGCCAAGAAT<br>R: GAGGTTTCCCGTGTTGAGTC	53.2<br>59.4	40
**VEGF-A**	*VEGFA*	F: CTACCTCCACCATGCCAAGT<br>R: GCAGTAGCTGCGCTGATAGA	59.3<br>60.0	40
**VEGF-B**	*VEGFB*	F: ACCAGAGGAAAGTGGTGTCAT<br>R: CCCATGAGCTCCACAGTCAAG	57.9<br>61.8	40
**VEGF-C**	*VEGFC*	F: CACGAGCTACCTCAGCAAGA<br>R: GCTGCCTGACACTGTGGTA	59.4<br>58.8	40
**VEGF-D**	*VEGFD*	F: CCTGAAGAAGATCGCTGTTC<br>R: GAGAGCTGGTTCCTGGAGAT	57.3<br>59.4	40
**VEGFR1**	*FLT1*	F: TCCGAAGCAAGGTGTGACTT<br>R: TATTGCCATGCGCTGAGTGA	57.3<br>57.3	40
**VEGFR2**	*KDR*	F: GAGGGGAACTGAAGACAGGC<br>R: GGCCAAGAGGCTTACCTAGC	61.4<br>61.4	40
**VEGFR3**	*FLT4*	F: GCACTGCCACAAGAAGTACCT<br>R: GCTGCACAGATAGCGTCCC	59.8<br>61.0	40
**PROX-1**	*PROX1*	F: CCCAGGACAGTTTATTGACCG<br>R: GGTTGTAAGGAGTTTGGCCCA	59.8<br>59.8	40
**ANGPT1**	*ANGPT1*	F: ACAACCTTGTCAATCTTTGCACT<br>R: TGCAAAACACCTTTTTGGGTTCT	57.1<br>57.1	40
**ANGPT2**	*ANGPT2*	F: ACCCCACTGTTGCTAAAGAAGA<br>R: CCATCCTCACGTCGCTGAATA	58.4<br>59.8	40
**ICAM1**	*ICAM1*	F: AGCTTCGTGTCCTGTATGGC<br>R: TTTTCTGGCCACGTCCAGTT	59.4<br>57.3	40
**HIF1a**	*HIF1A*	F: TTCCAGTTACGTTCCTTCGATCA<br>R: TTTGAGGACTTGCGCTTTCA	58.9<br>55.3	40
**TIE2**	*TIE2*	F: CTGAAAATGCTGACCGGGAC<br>R: GGCACTCAAGCCCTATCCAT	59.4<br>59.4	40

Primers were acquired from PrimerBank (public resource available at pga.mgh.harvard.edu/primerbank/) or designed using the primer designing tool of Primer-BLAST (available at www.ncbi.nlm.nih.gov/tools/primer-blast/).

Supplemental Figures

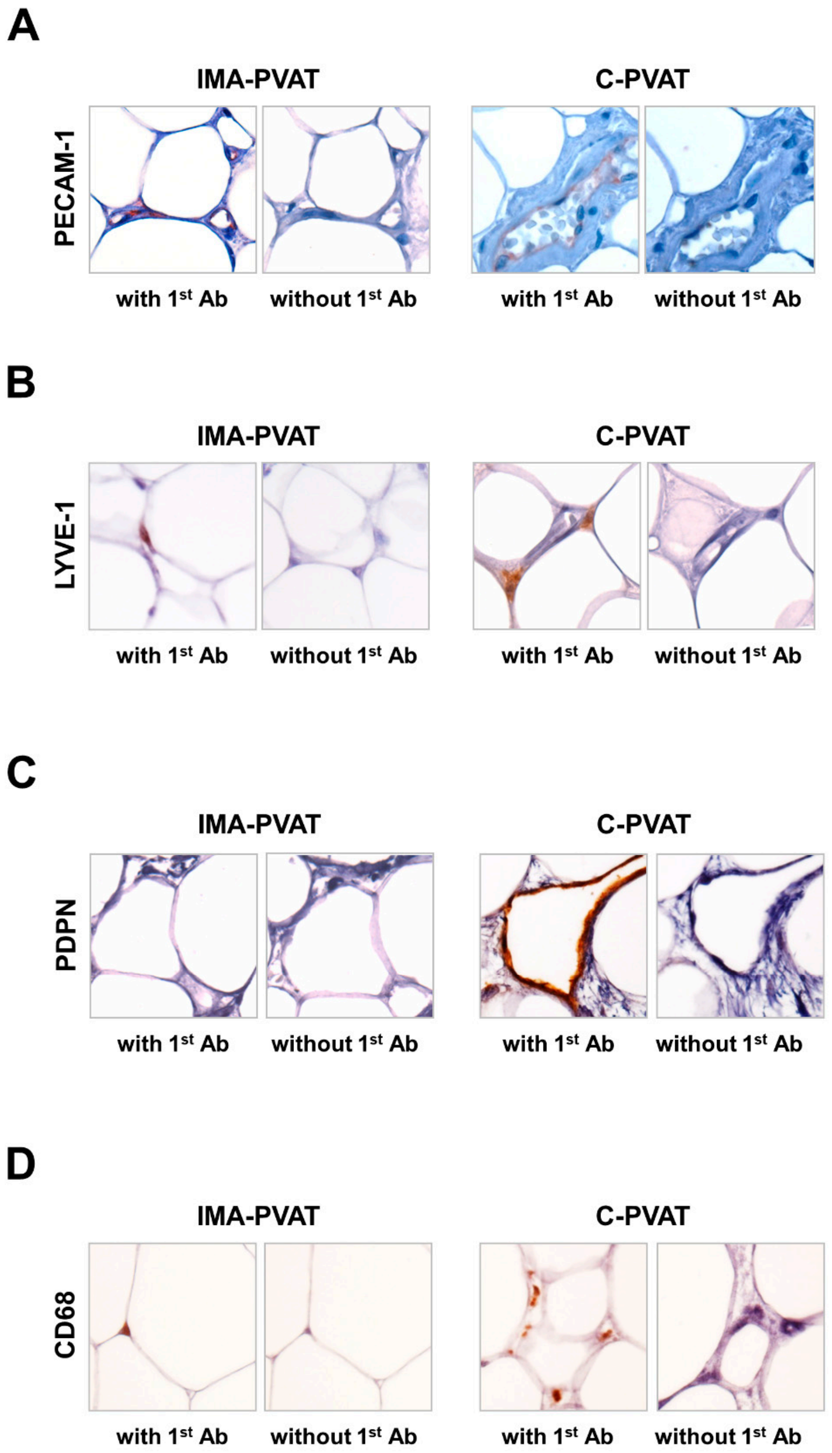

Figure S1. Immunohistochemical analysis of PVAT: negative controls. Immunohistochemical detection of PECAM-1 **(A)**, LYVE-1 **(B)**, PDPN **(C)** and CD68 **(D)** in IMA-PVAT and C-PVAT. Higher magnifications of findings after incubation with (left panels) and without (right panels) first antibodies are shown. Positive immunosignals are red (panel A) or brown (panels B-D).

	A	B	C	D	E	F	G	H	I	J	K	L
1	POS	POS	NEG	NEG	VEGF-C	VEGF-D	PECAM-1	VEGF-A	ANGPT1	ANGPT2	FGF2	LEP
2	POS	POS	NEG	NEG	VEGF-C	VEGF-D	PECAM-1	VEGF-A	ANGPT1	ANGPT2	FGF2	LEP
3	ADIPOQ	MMP1	MMP2	MMP9	TIMP1	TIMP2	CCL2	ICAM1	IL1A	IL1B	IL2	IL4
4	ADIPOQ	MMP1	MMP2	MMP9	TIMP1	TIMP2	CCL2	ICAM1	IL1A	IL1B	IL2	IL4
5	IL6	IL7	IL8	IL10	IL13	TNFA	TGFB1	IFN-gamma	SERPINE1	BLANK	BLANK	POS
6	IL6	IL7	IL8	IL10	IL13	TNFA	TGFB1	IFN-gamma	SERPINE1	BLANK	BLANK	POS

Figure S2. Cytokine antibody array map. Schematic representation of the target proteins and their localization on the membrane array, as shown in Figure 4A.

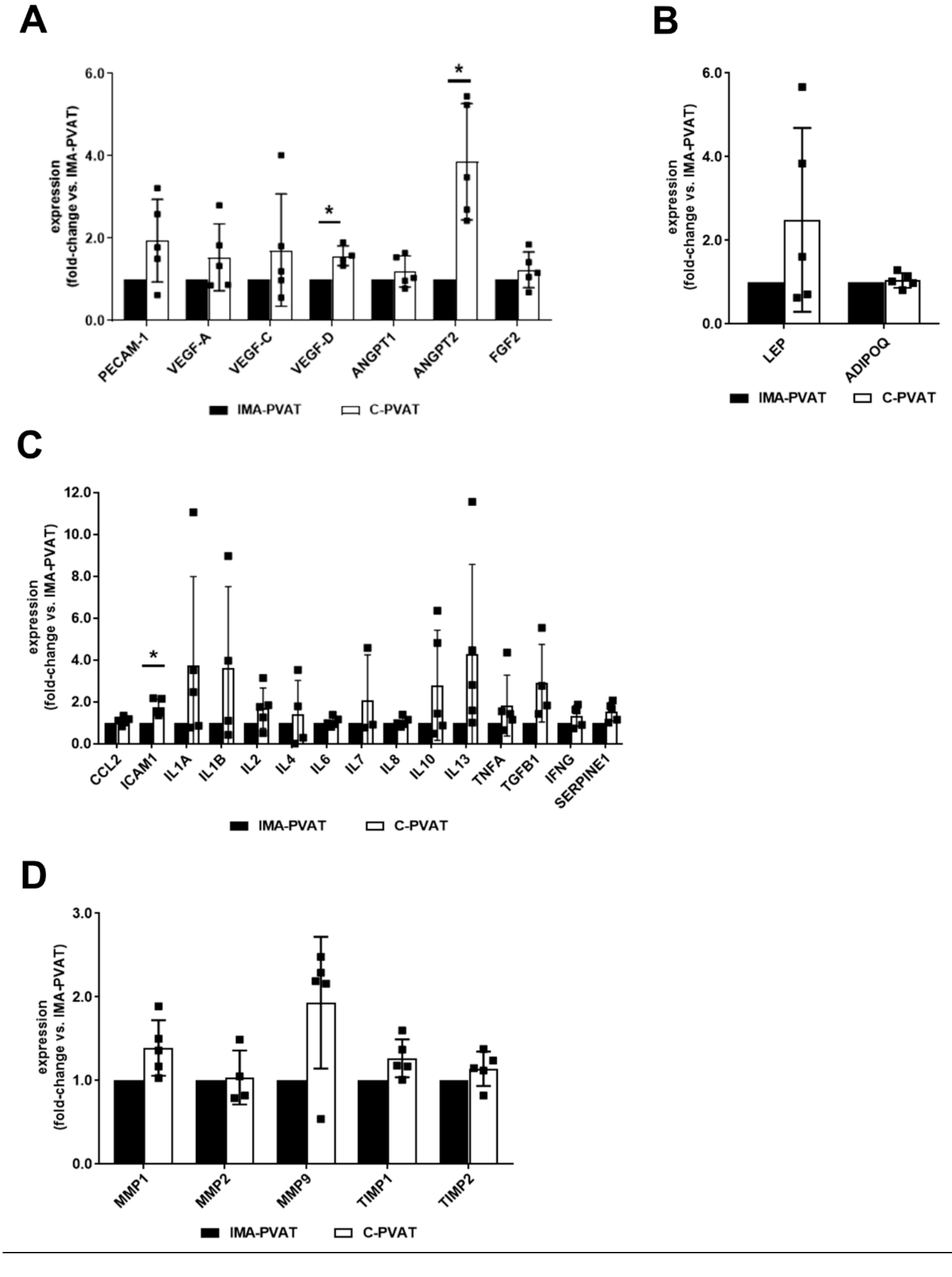

Figure S3. Quantitative analysis of protein expression in PVAT using cytokine antibody arrays. Quantitative analyses are shown for all 29 target proteins detected in conditioned medium from IMA-PVAT and C-PVAT of n=5 patients with CAD. Graphical presentation of protein expression is divided in four categories, namely (lymph)angiogenic growth factors **(A)**, adipokines **(B)**, inflammatory mediators **(C)**, and metalloproteinases and their inhibitors **(D)**. Individual values shown represent -fold changes of mean protein expression in IMA-PVAT. Error bars represent standard deviation. Statistical analysis was performed using Student's paired t-test. *P<0.05 vs. IMA-PVAT.

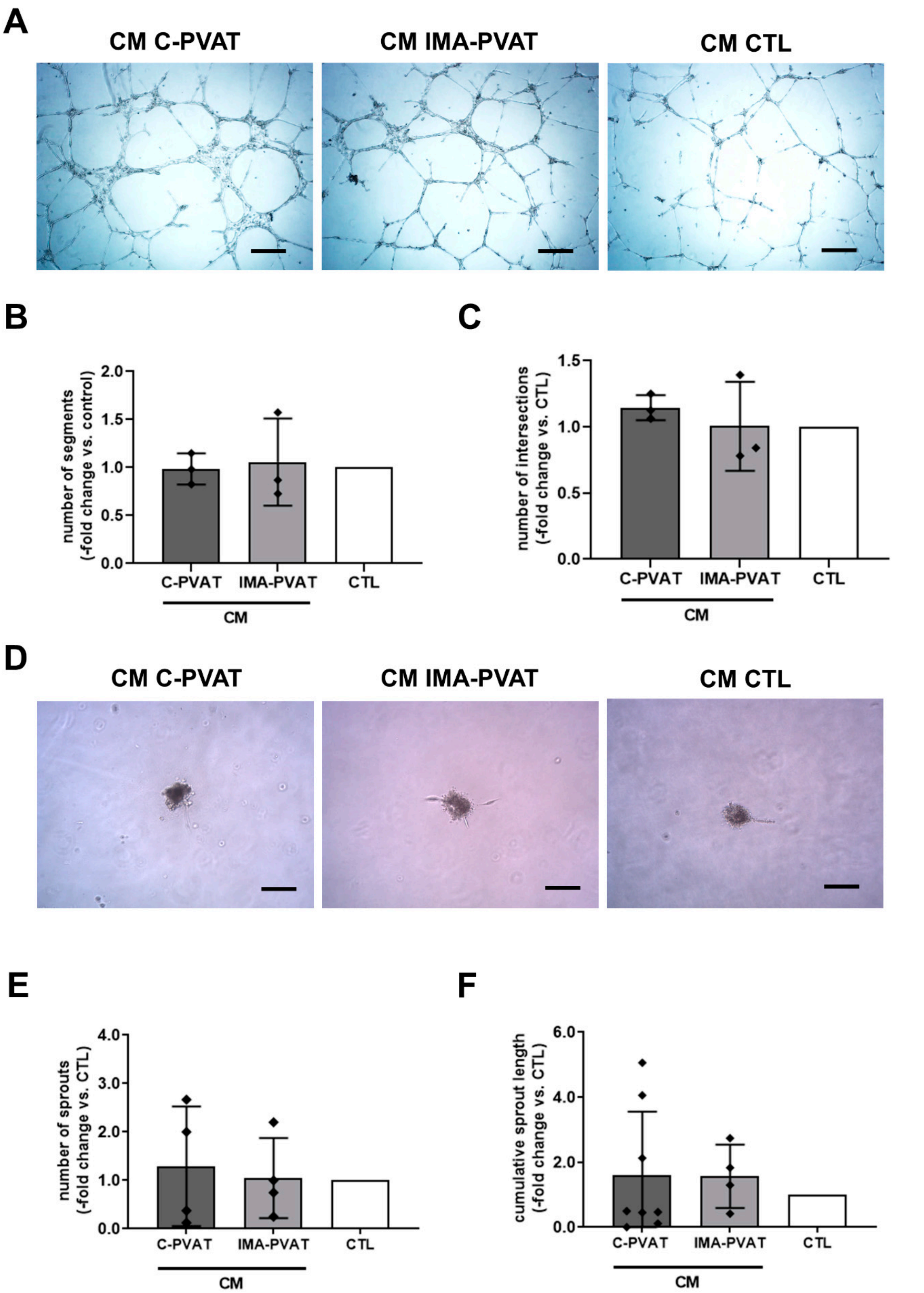

Figure S4. Analysis of paracrine effects of PVAT-derived conditioned medium on human dermal lymphatic endothelial cells. Representative images of human dermal lymphatic endothelial cell (HDLEC) tubes following incubation in matrigel™ **(A)** as well as the

quantitative analysis of the number of tube segments **(B)** and tube intersections **(C)** in n=3 independent experiments are shown. Representative images of HDLEC spheroids in collagen matrix **(D)** as well as the results after quantitative analysis of the number of sprout (per spheroid) **(E)** and the cumulative sprout length (µm) **(F)** in n=4-8 patients with CAD. In both experiments, HDLECs were incubated with conditioned medium from C-PVAT or IMA-PVAT or control (CTL), as described in the Methods.

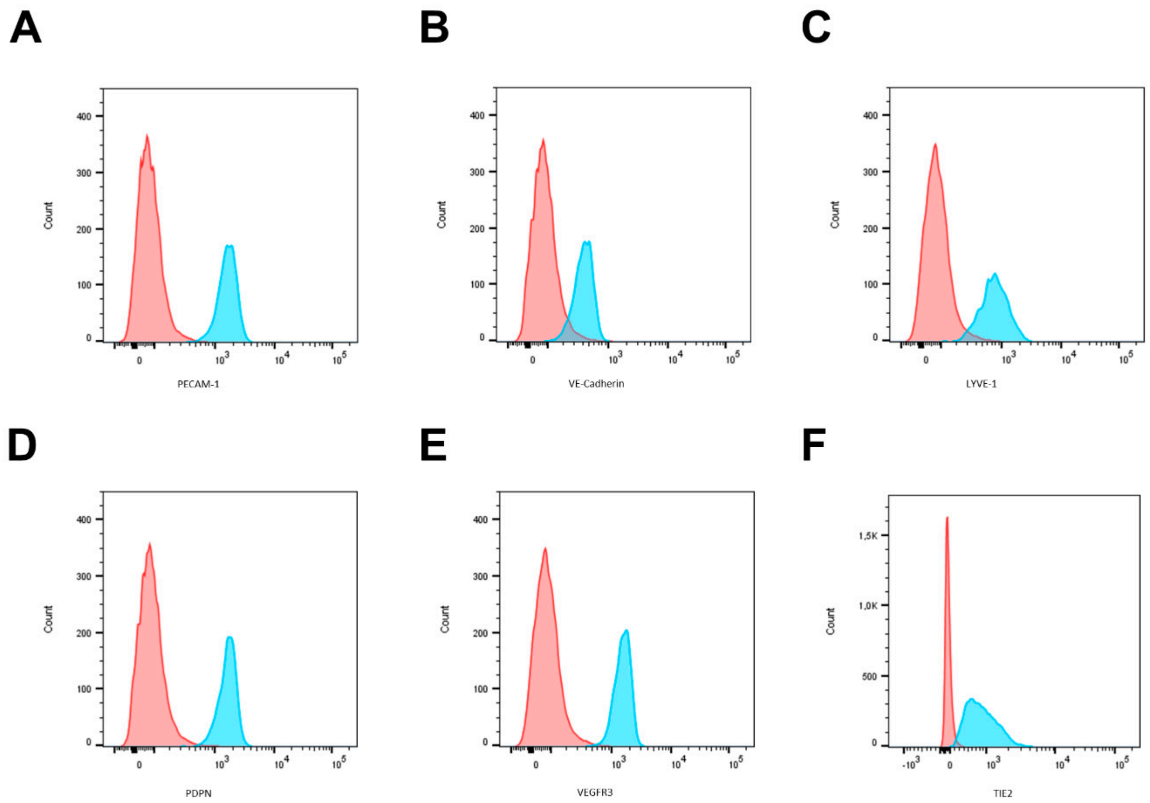

Figure S5. Flow cytometry analysis of (lymphatic) endothelial cell markers in HDLECs. Representative histograms showing the expression of lymphatic endothelial cell marker in HDLECs using flow cytometry. **(A)** PECAM-1, **(B)** VE-cadherin, **(C)** LYVE-1, **(D)** Podoplanin, **(E)** VEGFR3 and **(F)** TIE2. Graphs represent merged histograms of unstained control (red) and stained sample (blue).
